# Supplementary material for: Low-Bacterial Diet in Cancer Patients: A Systematic Review
Source: Nutrients. 2023 Jul 17;15(14):3171. doi: 10.3390/nu15143171 (PMC10385845; doi:10.3390/nu15143171)
Supplement: Supplementary file 1 [file nutrients-15-03171-s001.zip › nutrients-2500715-supplementary.pdf]

Supplementary File S1

Search Strategy

1. CENTRAL and DARE (The Cochrane Library) search strategy
#1 MeSH descriptor Agranulocytosis explode all trees<br>#2 MeSH descriptor Bacterial Translocation explode all trees<br>#3 MeSH descriptor Immunocompromised Host explode all trees<br>#4 (agranulocytosis or bacterial translocation or immunocompromised or cytopeni or immunocompromized or neutropeni or leukopeni or leucopeni or granulocytopeni)<br>#5 (#1 OR #2 OR #3 OR #4)<br>#6 MeSH descriptor Diet explode all trees<br>#7 (low near bacteria or low near microbia or minimal near bacteria or minimal near microbia or germ near poor or neutropenic or cooked or reduced near bacteria or sterile or clean)<br>#8 (diet or feeding or food or water or nutrition)<br>#9 (dietary restriction)<br>#10 (dietary near restriction)<br># 11 (#6 OR #7 OR #8 OR #9 OR #10)<br>#12 MeSH descriptor Bone Marrow Transplantation explode all trees<br>#13 MeSH descriptor Stem Cell Transplantation explode all trees<br>#14 (bone marrow transplantation) or (stem cell transplantation): ti,ab,kw<br>#15 (#5 OR #12 OR #13 OR #14)<br>#16 (#11 AND #15)<br>[ti,ab,kw = title or abstract or keywords]

2. PubMed search strategy
(("bone marrow transplantation"[MeSH Terms] OR "bone marrow transplantation"[Text Word] OR "cytopen*"[Text Word] OR "stem cell transplantation"[MeSH Terms] OR "stem cell transplantation"[Text Word] OR "agranulocytosis"[MeSH Terms] OR "agranulocytosis"[Text Word] OR "bacterial translocation"[MeSH Terms] OR "bacterial translocation"[Text Word] OR "immunocompromised host"[MeSH Terms] OR "immunocompromised host"[Text Word] OR "neutropeni*"[Title/Abstract] OR "leukopeni*"[Title/Abstract] OR "leucopeni*"[Title/Abstract] OR "granulocytopeni*"[Title/Abstract] OR "immunocompromized"[Title/Abstract] OR "immunocompromised"[Title/Abstract]) AND ((("sterile"[Title/Abstract] OR "clean"[Title/Abstract] OR "low bacteria*"[Title/Abstract] OR "low microb*"[Title/Abstract] OR "minimal bacteria*"[Title/Abstract] OR "minimal microb*"[Title/Abstract] OR "germ poor"[Title/Abstract] OR "cooked"[Title/Abstract] OR "reduced bacteria*"[Title/Abstract]) AND ("diet"[MeSH Terms] OR "diet"[Text Word] OR "feeding"[Title/Abstract] OR "dietar*"[Title/Abstract] OR "food*"[Title/Abstract] OR "nutrition"[Title/Abstract])) OR "dietary restriction"[Title/Abstract]))

3. EMBASE (Ovid) search strategy

#1: 'agranulocytosis':ti,ab,lnk,tn,tt,df,mn OR 'agranulocytosis'/exp
#2: 'stem cell transplantation':ti,ab,lnk,tn,tt,df,mn OR 'stem cell transplantation'/exp
#3: 'bone marrow transplantation':ti,ab,lnk,tn,tt,df,mn OR 'bone marrow transplantation'/exp
#4: 'bacterial translocation':ti,ab,lnk,tn,tt,df,mn OR 'bacterial translocation'/exp
#5: ‘immune deficiency’/exp
#6: (neutropeni$ or leukopeni$ or cytopeni$ or granulocytopeni$ or leucopeni$ or immunocompromized or immunocompromised)
#7: #1 OR #6
#8: ‘sterile' OR 'clean' OR 'low bacteria$' OR 'low microbia$' OR 'minimal bacteria$' OR 'minimal microbia$' OR 'germ poor' OR 'neutropenic' OR 'cooked' OR 'reduced bacteria$’
#9: ‘diet’/exp
#10: ‘diet$’ OR ‘water’ OR ‘feeding’ OR ‘food$’ OR ‘nutrition’
#11: #9 OR #10
#12: #8 AND #11
#13: ’dietary restriction$’
#14: #12 or #13
#15: #7 and #14
[mp= title, abstract, subject headings, drug trade name, original title, device manufacturer, drug manufacturer name; /exp = Emtree term]

4. CINHAL search strategy

#1: AB (sterile or clean or low bacteria$ or low microbia$ or minimal bacteria$ or minimal microbia$ or germ poor or neutropenic or cooked or reduced bacteria$)
#2: TI (sterile or clean or low bacteria$ or low microbia$ or minimal bacteria$ or minimal microbia$ or germ poor or neutropenic or cooked or reduced bacteria$)
#3: (#2 or #1)
#4: MH (diet+)
#5: AB (diet$ or water or feeding or food$ or nutrition)
#6: TI (diet$ or water or feeding or food$ or nutrition)
#7: (#6 or #5)
#8: (#7 or #4)
#9: (#8 and #3)
#10: (agranulocytosis+ or bacterial translocation+ or bone marrow transplantation+ or immunocompromised host+)
#11: AB (neutropeni$ or leukopeni$ or granulocytopeni$ or leucopeni$ or immunocompromized or immunocompromised or agranu- locytosis or bone marrow transplantation or stem cell transplantation or bacterial translocation or cytopen$)
#12: TI (neutropeni$ or leukopeni$ or granulocytopeni$ or leucopeni$ or immunocompromized or immunocompromised or agranu- locytosis or bone marrow transplantation or stem cell transplantation or bacterial translocation or cytopen$)
#13: (#12 or #11 or #10)
#14: AB (dietary restriction$)
#15: TI (dietary restriction$)
#16: (#14 or #15)
#17: (#16 or #9)
#18: (#17 and #13)
[AB = abstract; TI = title; MH = exact subject heading; + = explosion]

Risk of bias summary

Study	Item 1	Item 2	Item 3	Item 4	Item 5	Item 6	Item 7	Item 8	Item 9	Item 10	Item 11	Included	Comments
Ma et al. [5]	Y	Y	Y	Y	Y	Y	Y	Y	Y	Y	NA	Y	Meta-analysis
Ball et al. [15]	Y	Y	Y	Y	Y	Y	Y	Y	Y	Y	NA	Y	Meta-analysis
Sonbol et al. [7]	Y	Y	Y	Y	Y	Y	Y	Y	Y	Y	Y	Y	Meta-analysis

JBI Critical appraisal tool for systematic review and research syntheses

Legend: Y: yes; N: no; NA: not applicable / Items from Critical appraisal tool for systematic review and research syntheses: 1.Is the review question clearly and explicitly stated?; 2.Was the search strategy appropriate?; 3.Were the sources and resources used to search for studies adequate?; 4.Were the criteria for appraising studies appropriate?; 5.Was critical appraisal conducted by two or more reviewers independently?; 6.Were there methods to minimize errors in data extraction?; 7.Were the methods used to combine studies appropriate; 8.Was the likelihood of publication bias assessed?; 9.Were recommendations for policy and/or practice supported by the reported data?; 10.Were the specific directives for new research appropriate?

Study	Item 1	Item 2	Item 3	Item 4	Item 5	Item 6	Item 7	Item 8	Item 9	Item 10	Included	Comments
Jakob et al. [18]	Y	Y	Y	Y	Y	Y	Y	Y	Y	Y	Y	Case control

JBI Critical appraisal tool for case control studies

Legend: Y: yes / Items from JBI Critical appraisal tool for case control studies: 1.Were the groups comparable other than the presence of disease in cases or the absence of disease in controls?; 2.Were cases and controls matched appropriately?; 3.Were the same criteria used for identification of cases and controls?; 4.Was exposure measured in a standard, valid and reliable way?; 5.Was exposure measured in the same way for cases and controls?; 6.Were confounding factors identified?; 7.Were strategies to deal with confounding factors stated?; 8.Were outcomes assessed in a standard, valid and reliable way for cases and controls?; 9.Was the exposure period of interest long enough to be meaningful?; 10.Was appropriate statistical analysis used?

Study	Item 1	Item 2	Item 3	Item 4	Item 5	Item 6	Item 7	Item 8	Included	Comments
Toenges et al. [29]	Y	Y	U	Y	Y	N	Y	Y	Y	Limitations in the methodology of the study (survey)
Fang et al. [24]	U	N	Y	N	Y	Y	N	U	Y	Not validated questionnaire used
Heng et al. [12]	Y	Y	Y	Y	Y	Y	Y	Y	Y	Retrospective study, convenience sample
Brown et al. [30]	Y	N	Y	N	N	N	Y	Y	Y	Limitations in the methodology of the study (survey)
Gupta et al. [31]	U	Y	N	Y	N	N	U	U	Y	Limitations in the methodology of the study (survey)
Jeon et al. [16]	Y	Y	Y	N	Y	Y	Y	Y	Y	Not validated questionnaire used
Peric et al. [1]	Y	Y	U	N	Y	N	U	N	Y	Not validated questionnaire used
Carr and Halliday [19]	Y	Y	U	N	U	U	N	U	Y	Not validated questionnaire used

JBI Critical appraisal tool for analytical cross-sectional studies

Legend Y: yes; N: no; U: unclear; NA: not applicable / Items from Critical appraisal of analytical cross-sectional studies: 1.Were the criteria for inclusion in the sample clearly defined?; 2.Were the study subjects and the setting described in detail?; 3.Was the exposure measured in a valid and reliable way?; 4.Were objective, standard criteria used for measurement of the condition?; 5.Were confounding factors identified?; 6.Were strategies to deal with confounding factors stated?; 7.Were the outcomes measured in a valid and reliable way?; 8.Was appropriate statistical analysis used?

Table S1. The baseline characteristics of all included studies.

First author	Date (y)	Design	Setting	Number of (n)	participants	Outcome measures
				Study group	Control group	
Ma et al. [5]	2022	Meta-analysis	NA	113	106	Infection and Mortality
Toenges et al. [29]	2021	Cross-sectional	in and out	28	//	Infection
Jakob et al. [18]	2021	Case control	in	1043	1043	Infection, Mortality and QoL
Fang et al. [24]	2020	Cross-sectional	in	18	//	//
Ball et al. [15]	2019	Meta-analysis	NA	113	106	Infection
Heng et al. [12]	2019	Cross-sectional	in	79	75	Infection
Brown et al. [30]	2019	Cross-sectional	in and out	20	//	//
Gupta et al. [31]	2019	Cross-sectional	in	229	//	//
Jeon et al. [16]	2018	Cross-sectional	in and out	123	//	//
Peric et al. [1]	2018	Cross-sectional	in	90	//	//
Sonbol et al. [7]	2015	Meta-analysis	NA	88	85	Infection and Mortality
Carr and Halliday [19]	2015	Cross-sectional	in and out	110	//	//

Legend: y: years; n: number; in: inpatient; out: outpatient; NA: not applicable

Table S2. The application of LBD.

Study	Design	Intervention	
		Study group	Control group
Ma et al. [5]	Meta-analysis	Patients received the neutropenic diet, low bacterial diet and cooked diet	Patients received raw diet, unrestricted diet, FDA-approved food safety guidelines and regular hospital diet
Toenges et al. [29]	Cross-sectional	Patients received the low bacterial diet	//
Jakob et al. [18]	Case control	Patients received no raw fruits and vegetables (cooked diet)	Patients received diet without restrictions
Fang et al. [24]	Cross-sectional	Patients received the neutropenic diet,	//
Ball et al. [15]	Meta-analysis	Patients received the neutropenic diet, low bacterial diet and cooked diet	Patients received raw diet, unrestricted diet, FDA-approved food safety guidelines and regular hospital diet
Heng et al. [12]	Cross-sectional	Patients received the neutropenic diet	Patients received diet without restrictions
Brown et al. [30]	Cross-sectional	Patients received the neutropenic diet	//
Gupta et al. [31]	Cross-sectional	Patients received the neutropenic diet	//
Jeon et al. [16]	Cross-sectional	Patients received the neutropenic diet	//
Peric et al. [1]	Cross-sectional	Patients received the neutropenic diet	//
Sonbol et al. [7]	Meta-analysis	Patients received the neutropenic diet and cooked diet	Patients received raw diet, unrestricted diet, FDA-approved food safety guidelines and regular hospital diet
Carr and Halliday [19]	Cross-sectional	Patients received the neutropenic diet	//
